# Tailoring the adhesion properties of thin polymeric films using additives: an AFM study

**DOI:** 10.1039/d5na00956a

**Published:** 2026-01-14

**Authors:** Sebastiaan Haartsen, Inga Wille, Harald Jasper, Harold J. W. Zandvliet, Johannes Aprojanz, Pantelis Bampoulis

**Affiliations:** a Physics of Interfaces and Nanomaterials Group, MESA+ Institute for Nanotechnology and University of Twente PO Box 217 7500AE Enschede The Netherlands s.haartsen@utwente.nl p.bampoulis@utwente.nl; b ACTEGA Metal Print GmbH Mielestrasse 13 31275 Lehrte Germany

## Abstract

This study investigates the modification of adhesive properties in UV-cured thin films, commonly used in many fields ranging from protective coatings to primer layers for printing. We incorporated two types of additives into a 12 µm thick polymer film: one additive containing silicone-modified polyethers and another additive containing silicone-free modified polyethers. Our findings indicate that both additives segregate towards the film's surface, altering the surface properties without affecting the bulk. Using atomic force microscopy, we measured the adhesive work from force–distance curves, observing improved adhesive properties up to an optimal concentration of 10 wt%. Beyond this concentration, the film's adhesion plateau, with excess additives assimilating into the film's bulk which we interpret as being consistent with a change in the near-surface polymer ordering. Concentration-dependent measurements suggest a change in nanomechanical response above 10 wt%. This indicates that the films above this concentration undergo a drastic change, which we attribute to either capillary interaction, molecular ordering or additional crosslinking between the additive and base polymer mixture. Our results provide a deeper understanding of polymeric surface modification, which is paramount for flexographic printing of metallic surfaces using 2D flakes and thin polymer films.

## Abstract 2

Using cross-sectional atomic force microscopy, we investigated how different additives modify the adhesive properties of UV-cured polymer films used in coatings and printing primers.

## Graphical abstract

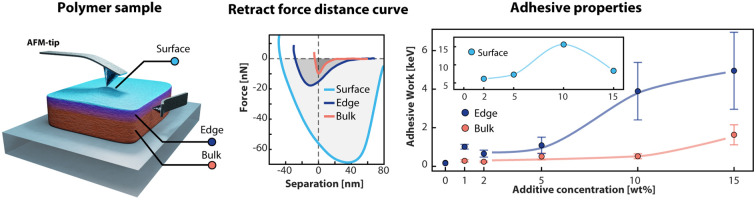

## 1 Introduction

Micrometre-thin films synthesised from polymers are utilised for multiple applications, ranging from protective layers in drug delivery^1^ to catalysts in solar cells to improve optical responses and efficiency.^2,3^ In the graphic arts, specifically printing, thin adhesive films serve as both protective coatings and primer layers.^4^ The films are tailored to specific mechanical properties, like adhesion and stiffness, to improve the durability of printed images and ensure precise ink transfer to a patterned substrate.^5–7^ Specifically for two-dimensional (2D) material flake printing, adhesive polymeric films enable the transfer of metal flakes onto a functionalised layer, structured for high-resolution deposition.^8^ The film's adhesiveness, optimised beyond that of both the substrate and ink ‘donor’ roller, ensures that the metal flakes in the ink adhere exclusively to the film's surface. This process leverages both physisorption and chemisorption, depending on the interaction strength desired.^9,10^

Adding an additive to these polymeric films can significantly alter the properties by catalysing or decelerating reactions, such as UV curing,^11,12^ or by modulating the rate of chemical degradation to improve the film quality or substrate durability.^13,14^ Low concentrations of additives can already significantly impact the film but may be difficult to detect using space-averaging surface techniques.^15,16^ Therefore, more surface sensitive techniques are required to accurately measure surface modifications due to the additive.^17^ On the other hand, high additive concentrations can lead to film saturation and increased costs.^18^ This necessitates optimisation in the film's chemical composition to minimise material usage while maintaining optimal surface functionalisation. The effect of an additive on the mechanical properties of a film varies with both the type and concentration of the additive. Mechanical properties such as adhesion and indentation can increase or decrease depending on these parameters.^10,19,20^

Our study examines the modification of the adhesive properties of UV-cured thin films, incorporating two types of additives into a 12 µm film: the first additive consists of polyethers modified with silicone and the other additive contains modified polyethers without silicone. Using atomic force microscopy (AFM) and cross-section AFM, we found that the additives segregate toward the film–surface interface, altering the surface properties. Force–distance analysis combined with quantitative nanomechanical mapping (QNM), allows us to resolve the mechanical properties of the polymeric surface with a spatial resolution in the nanometer range.^21^ Using QNM, we measured the adhesive properties of the films along with indentation.^22–24^ We note here that cross-sectional AFM allows for characterisation of the mechanical properties normal to the surface, capturing both the edge and bulk of the polymeric film to study the modifying capabilities of the additives. Using both the topography and nanomechanical properties of the film, we are able to locate the edge, close to the film's top surface, and the bulk, between the edge and the film–substrate interface. Results and methods from this study can be used to create more sustainable polymeric films with highly functional surface properties tailored for the specific transfer of 2D ink pigments or 2D materials.

## 2 Methods

The base polymer film mixture consists of a long- and short-aliphatic polyether diacrylate to balance the stiffness and stickiness of the film, together with a mixture of type 1 and type 2 photoinitiators that uniformly cure the film's surface and bulk. After mixing the components for about 10 minutes at 50 °C, a few droplets of the liquid are deposited on a flexible polyethylene terephthalate (PET) substrate. The liquid is distributed homogeneously over the PET substrate using a coating or roller bar with a sample distance of 12 µm. The samples are UV-cured for a few seconds to form the polymer thin film. Surface tension measurements using the pendant drop method and a drop shape analyser (Krüss DSA30E) give a surface tension of 38 mN m^−1^. The additives are added to the liquid blend before mixing. The selected additives are suitable for UV-curable polymeric films and are commercially available from BYK-Chemie GmbH. BYK-333 contains polyether-modified polydimethylsiloxane (silicone) and decreases the surface tension of the polymer film from 35 mN m^−1^ to 21 mN m^−1^ for concentrations above 0.5 wt%. The BYK-3535 additive is hyperbranched and contains modified silicone-free polyethers.^16^ The surface tension of this additive is 35 mN m^−1^ and similar to the base polymer mixture. Using this sample fabrication method, we prepared 7 unique films with additive concentrations that plateau the surface tension (>0.5 wt%) of the film.

The concentration of the additive is determined from the mass fraction of the additive and the total liquid mixture and is calculated in wt%. The additive concentration in the mixtures are 0 wt% (polymer base mixture), 1 wt%, 2 wt%, 5 wt%, 10 wt% and finally 15 wt%. Concentrations exceeding 15 wt% are outside the formulation range for standard flexographic printing processes and therefore beyond the scope of this study. After a UV-curing step, the finished samples show a homogeneous spreading over the PET substrate and no phase separation. The polymer film and substrate are transparent, but can be distinguished from the substrate using an optical microscope for AFM surface- and cross-sectional spectroscopy. The films are cut, using scissors that are cleaned with isopropanol, into small strips and glued on the sample holder of the AFM (Bruker Dimension Icon), with silver paste for structural support. The PET-substrate provides a base to ensure that the polymer film is aligned and secured in the correct position (SI Fig. 1). The roller bar direction is aligned to the surface, and the cross-sectional area is positioned normal to the sample holder. Before and after each measurement, the mechanical properties of the AFM-tips (PPP-FMR from Nanosensors, spring constant: *C* = 4 N m^−1^, resonance frequency: *f* = 75 kHz and a radius of curvature at the apex of 7 nm) are measured using a stiff sapphire sample. Due to the high probability of polymers attaching to the AFM tip, an additional calibration step is implemented. Polymer contamination of the tip is identified by force–distance analysis on a calibration sample (highly oriented pyrolytic graphite (HOPG)) with known mechanical properties (SI Fig. 2). Deviations of the force distance curves from the initial reference curve are taken as a signature of tip contamination. In such cases, we either clean the tip by continued scanning on the calibration sample or, if the tip cannot be restored, replace it with a new tip of the same specifications.

The AFM scanning location is determined by an optical microscope, which allows for an accurate approach to the (cross-sectional) area of interest on the film. We use non-contact, tapping mode to reduce sample- and tip deterioration during the measurements with a driving frequency of 2 kHz for all samples in ambient conditions (20 °C and 45% RH). The feedback loop is controlled by a maximum force of ≈10 nN with which we push the tip onto the sample. This allows for a good tip–sample contact. Furthermore, continued measurements on the same locations show no evidence of irreversible damage to the films. In tapping mode, we can measure the topography, adhesion and indentation of the sample simultaneously in a grid fashion. This grid mapping mode uses force–distance analysis to calculate the nanomechanical properties of the sample into a spatial 2D map.

## 3 Results

**Fig. 1:**
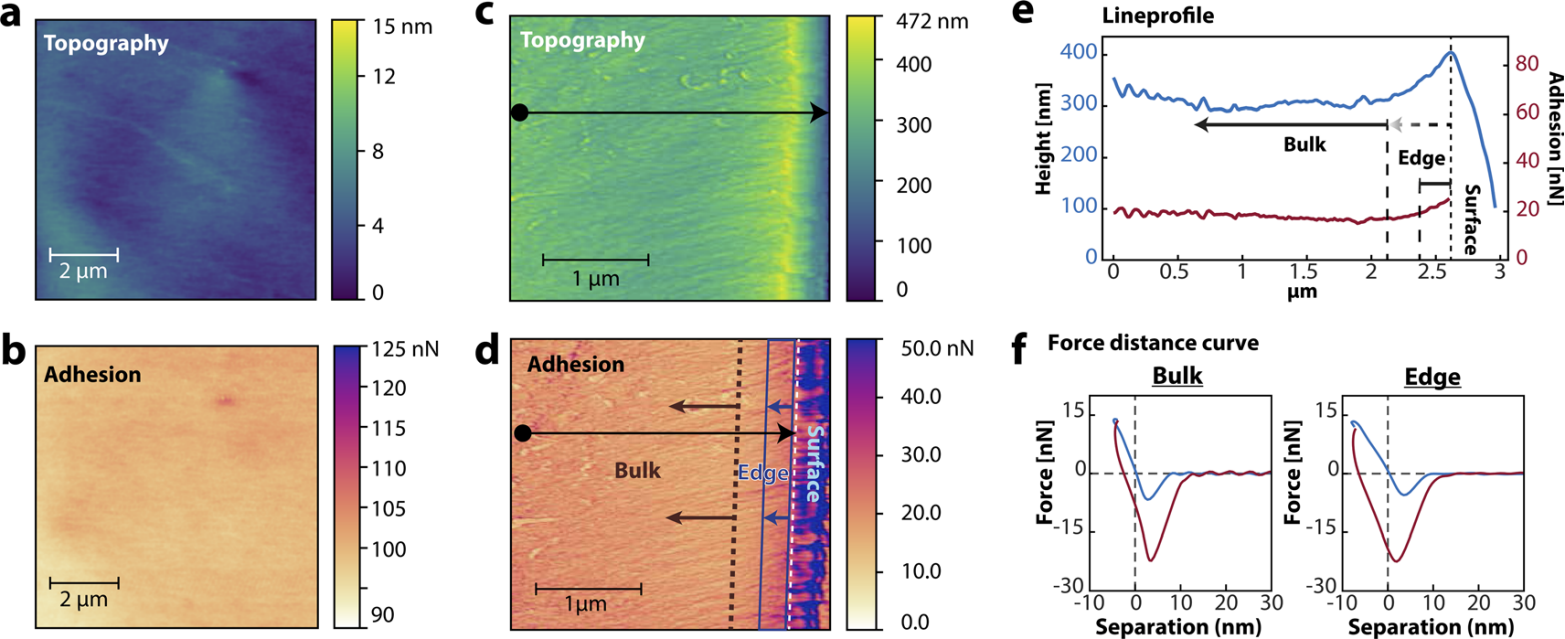
AFM grid spectroscopy results from the polymer thin film without an additive. (a) Topography and (b) adhesion maps of the polymer film surface without an additive. (c) Cross-sectional topography and (d) adhesion map of the polymer film without an additive. The arrows indicate edge (blue) and bulk (brown) areas of the cross-sectional surface. (e) Line profile of the cross-sectional maps, indicated by the black arrow in (c) and (d). The sudden decrease in the topography corresponds to the top surface of the film. (f) Force–distance curves, captured on the cross-sectional area in the bulk (left) and edge (right) of the film. The approach curve is shown in blue, and the retract curve is shown in red.

Fig. 1a and b show the topography and adhesion maps (64 µm^2^), respectively, obtained from a grid spectroscopy measurement of the polymer film's surface without an additive. The surface measurements reveal a film RMS (Root Mean Square) roughness of 3.8 nm and average surface adhesion of 100 nN ± 2 nN with a maximum of 111 nN. The cross-sectional measurements are shown in Fig. 1c and d. Fig. 1c shows the topography (9 µm^2^) and Fig. 1d displays the corresponding cross-sectional adhesive force map, measured simultaneously. On the right side of the map, the edge of the cross-section is apparent from the pronounced drop in height and the simultaneous increase in adhesion. The sharp change in adhesive interactions beyond the edge can be understood from the tip–sample geometry. As the tip moves past the surface line, the apex is no longer in contact, but the side of the AFM tip remains in contact with the sample. This side contact has a larger effective contact area, leading to an increased adhesive force. The RMS roughness of the cross-sectional topography can be separated into an edge region (0 to ≈250 nm from the surface) with ≈17 nm roughness and a bulk region (>250 nm from the surface) with ≈15 nm roughness. Similarly, the average adhesion at the edge is ≈19 nN, compared to ≈18 nN in the bulk.

From the line profile in Fig. 1e, it is apparent that the adhesive properties of the polymer film without an additive show small deviations across the cross-sectional measurement. This indicates that the film's composition is quite uniform. The force–distance curves in Fig. 1f, show an approach (blue) and retract (red) curve and are measured in the bulk and edge areas of the cross-sectional map of Fig. 1d. Force–distance analysis reveals tip–sample interactions and is used to identify dominant forces^25^ of the tip–sample interactions.^9,26^ The sudden snap-in due to van der Waals forces is visible in the approach (blue curve) regime as the tip–sample distance reduces to 0. After reaching the maximum force of ≈10 nN, the AFM-tip is retracted (red curve), and we observe a negative tip–sample separation at 0 nN, indicating a non-elastic deformation, as is common for soft materials due to energy dissipation during pressing. The long-range forces are captured relatively far from the sample after the minimum attractive force. Beyond this, the tip–sample distance increases while the force decreases, indicating that the tip loses contact with the sample and experiences no more force. In this regime, electrostatic forces^27^ along with polymer–tip interactions, are dominant.^28^

**Fig. 2:**
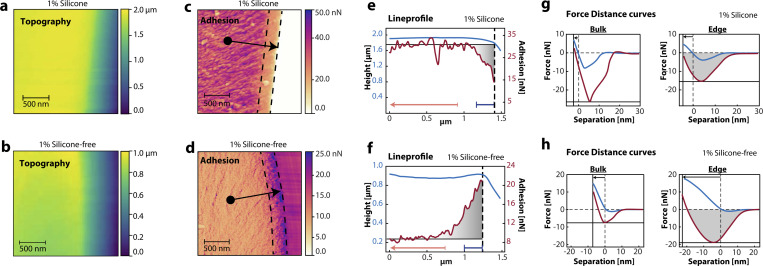
(a) Cross-sectional topography measurements of a polymer film with 1 wt% additive containing silicone. (b) Cross-sectional topography measurements of a polymer film with 1 wt% additive containing the silicone-free additive. (c) Cross-sectional adhesion map measured simultaneously with a. The dotted lines indicate the edge area. (d) Cross-sectional adhesion map measured simultaneously with (b). (e) Line profile of the adhesion, indicated by the arrow in (c). The film adhesion decreases from the bulk (left of the orange arrow) to the edge (blue arrow), due to the presence of the additive. (f) Line profile of the adhesion, indicated by the arrow in (d). Showing an increase in the film's adhesion from bulk to edge. (g) Force–distance curves (approach in blue and retract in red) of the film with 1 wt% additive containing silicone. Relative changes in the edge compared to the bulk are visible for adhesion and indentation. (h) Force–distance curves of the film with 1 wt% silicone-free additive. Relative changes in the edge compared to the bulk are visible for adhesion and indentation in both films (highlighted by the horizontal and vertical lines, respectively).

Adding an additive to the liquid blend changes the chemical composition of the polymeric thin film before UV-curing and, as we will show, the film's mechanical properties after UV-curing. This results in modified adhesive properties while sample thickness and roughness remain comparable to the sample without additives. Fig. 2 shows cross-sectional measurements of polymer thin films containing 1 wt% additive. Fig. 2a, c and e show the results from the additive containing silicone polyethers, and Fig. 2b, d and f correspond to the silicone-free additive.

Compared to the results shown in Fig. 1, we see differences as the adhesive force from the bulk towards the edge of the film can drastically change depending on the type of additive mixed in the blend. This change is apparent due to contrast changes in Fig. 2a and b. The modification of the adhesive force, when moving from the bulk towards the edge, is further recorded by the line profile in Fig. 2c and d. These measurements show that the polymers in the additive segregate towards the top surface.^29–32^ We hypothesise that this segregation takes place while the mixture is still liquid, between the deposition of the blend and UV-curing. After UV-curing, the monomers are linked, the film is solid, and the polymers are ‘frozen’, reducing surface segregation significantly.

When we add an additive containing silicone in the mixture, we measure a decrease in the adhesive interactions, while a silicone-free additive results in an increase in the adhesive interactions. To investigate this difference further, we provide the force–distance curves from the bulk (left) and edge (right) in Fig. 2e and f, to inspect tip–sample interactions. The approach is plotted in blue, and the red curve shows the retraction, comparable to Fig. 1f. The minimum adhesive force of the retract curves differs between the bulk and edge as we move closer to the surface for both types of additives. Moreover, the effect of the additive is only present near the surface interface, while bulk properties show reduced long-range interactions (>5 nm). Long-range tip–sample interactions include electrostatic forces or polymeric detachment and/or stretching, the latter being more apparent when measuring acrylic samples.^26,33,34^ These results show that we can capture the effect of the additive on the polymeric thin film and that both additives segregate towards the surface. However, the additive with silicone reduces the adhesive properties, while the silicone-free additive improves the adhesive properties of the film, highlighted by the lower adhesion minimum and longer-ranged interactions.

These results show that both types of additives migrate towards the surface and thereby modify the surface energy of the mixture at this location. This change in surface energy directly affects the capillary interaction between the AFM tip and the sample. Since the force–distance measurements are performed at ≈45% RH, a water meniscus forms between tip and sample, giving rise to a capillary force that contributes both to the adhesive force minimum and to the attractive tail during tip retraction as the meniscus collapses.^35^ Because the relative humidity and tip properties remain constant, differences in adhesion between the two different formulations reflect primarily changes in the sample surface energy induced by the additives. Moreover, polymeric contributions are nevertheless present and superimposed on this capillary background. The force–distance spectroscopy measurements show a tail in the retract curve extending over tens of nanometres, which we could be a consequence of stretching of polymers before the tip loses contact.^9,36,37^ Due to the heterogeneous nature of the sample, individual chain events cannot be resolved. Instead, many chains detach collectively, giving rise to a smooth long-range attractive force. Once the tip is sufficiently far from the surface, all polymers detach and the force returns to zero.

Conventional adhesion measurements cannot fully capture the polymeric interactions between the tip and sample. The adhesion is determined from the minimum of a force–distance curve, but this single value contains no information about the long-range attractive contributions that arise from both capillary forces (due to a water meniscus) and the stretching and detachment of polymers. These interactions are captured when we calculate the adhesive work from a force–distance curve. For the adhesive work, we calculate the area of the retract curve in the attractive regime below 0 nN, shown in grey in Fig. 2e and f. The tail features of the curve will then result in a larger adhesive work, reflecting increased long-range interactions between the tip and sample, arising from a combination of capillary forces and polymeric bridging. As the tip retracts, the adsorbed chains are stretched and eventually detach. The force on the cantilever remains non-zero until all polymers have detached and the meniscus has ruptured, far away from the sample, at which point no further force is measured. Therefore, we propose that the adhesive work is more suitable for characterising polymer thin films than the adhesion minimum alone, as it captures the full range of tip–sample interactions, including both capillary and polymeric contributions. In addition, because the force–distance curves are acquired at a fixed maximum load, local variations in film structure and mechanical compliance also modulate the adhesive work *via* changes in contact area and meniscus geometry. Therefore, we propose that the adhesive work is more suitable for characterising polymer thin films, as tip–sample interactions are captured more accurately compared to other mechanical properties. Due to the enhancing properties and comparable surface energy of the additive with respect to the base polymer mixture, we continue our study with the silicone-free additive.

**Fig. 3:**
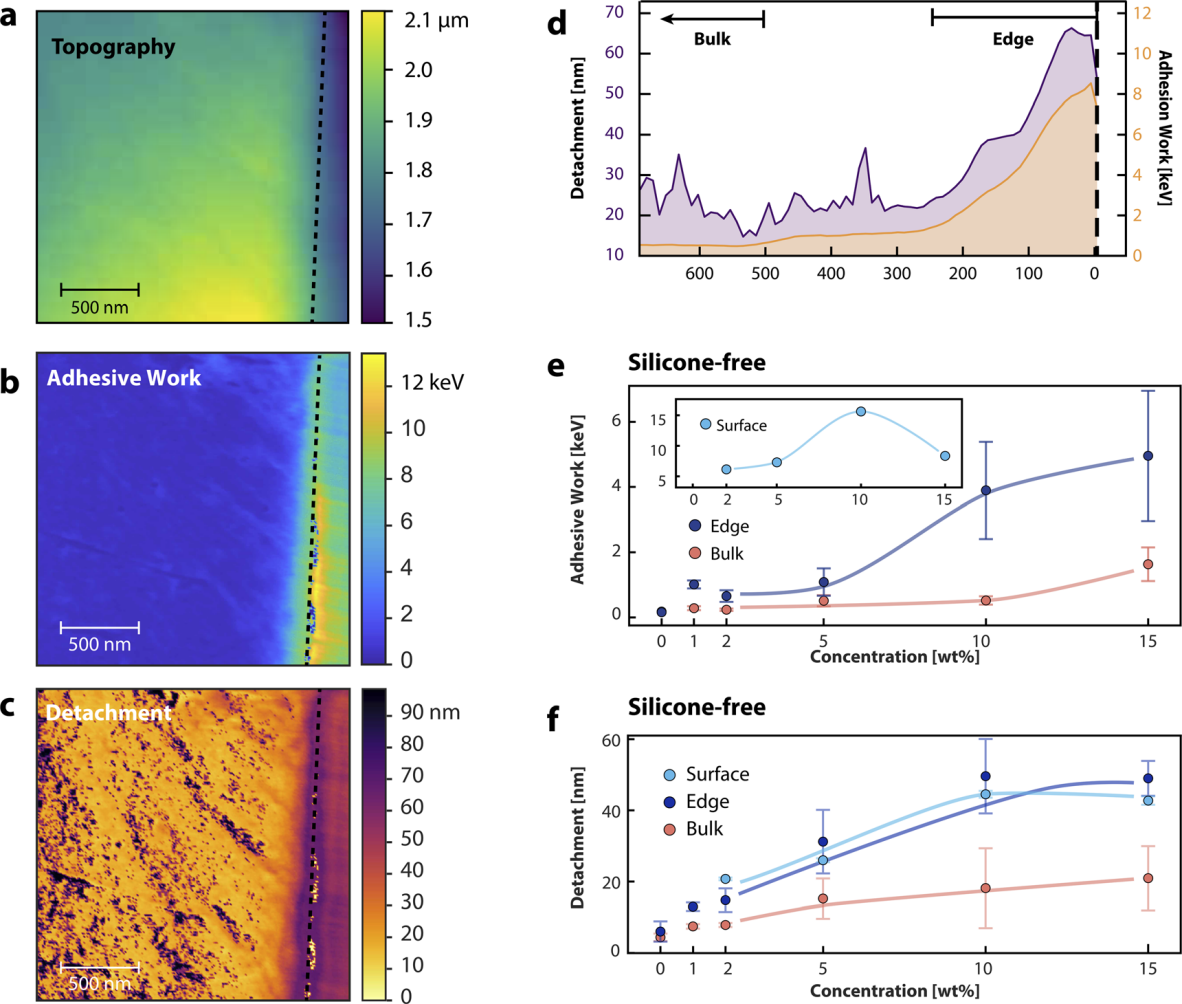
(a) Cross-sectional topography, (b) adhesive work and (c) detachment length map of a polymer thin film containing 10 wt% silicone-free additive. (d) Line profile corresponding to the maps shown in (a), (b) and (c). (e) Adhesive work as a function of concentration from the cross-sectional measurement, separating bulk (orange) and edge (dark blue) measurements. The inset shows the concentration dependence of the surface measurements (light blue). (f) Detachment length as a function of concentration for bulk, edge and surface. The lines presented in the graphs are guides to the eye.

When we increase the silicone-free additive concentration from 1 wt% to 10 wt%, the adhesive properties of the film are improved even further. Fig. 3 shows the measured maps of the topography in a, and the calculated adhesive work map in b. Compared to the 1 wt% film in Fig. 2b, we see similar features, however, the effect is enhanced. The contrast difference in Fig. 3b indicates that the adhesive work gradually increases from the bulk to the edge, while the bulk remains almost unaffected. The line profile in Fig. 3d highlights the gradual increase in adhesive work, moving from the bulk (1 keV) to the edge (6–8 keV) with a maximum of 12 keV close to the surface of the film.

In Fig. 3e we show the adhesive work as a function of the concentration of the silicone-free additive. The edge is shown in dark blue and the bulk in orange. Already at low percentages of additive, a deviation of the adhesive work between the bulk and edge is visible relative to the measurement without additive. The adhesive work of the bulk remains almost constant while the adhesive work of the edge increases up to 10 wt%. For comparison, we performed the same analysis on film's surface, shown in the inset of Fig. 3e. The adhesive work of the film at the surface reaches a maximum at about 10 wt% additive concentration, in line with the cross-sectional AFM measurements. When the concentration of the additive is increased to 15 wt%, the adhesive work in the bulk catches up with the edge and surface. From the latter, we conclude that the edge is saturated with the additive and excess polymers are assimilated into the bulk.

Fig. 3c shows a map of the sample–tip detachment length for the 10 wt% film. This detachment length is defined as the distance in the retract curve between the minimum adhesion force and the point at which the force returns to 0 nN and the tip is out of contact. In this attractive (negative) regime, the tip is retracted while multiple polymers are attached to the tip, and a capillary meniscus is present. This detachment length thus reflects the range over which attractive tip–sample interactions persist, with a contribution from the capillary meniscus and a long-range contribution from polymer bridging. Because the silicone-free formulations have comparable surface energies, the capillary contribution is expected to be similar, and thus differences in the spectra are expected to reflect mainly polymer–tip interactions. The contrast difference in the map shown in Fig. 3c indicates that the edge exhibits a longer detachment length compared to the bulk. Fig. 3f shows the detachment length for bulk, edge and surface as a function of additive concentration. Increasing the additive concentration results in longer detachment lengths, possibly due to the larger number of polymers attaching to the tip. Above 10 wt%, the detachment length saturates for the edge and surface.

In contrast to the bulk and edge measurements for both detachment length and adhesive work, adhesive work surface measurements (inset Fig. 3e) show a decrease of the adhesive work between 10 and 15 wt%, indicating that polymeric interactions are reduced as the film surface is saturated with the additive at higher concentrations. However, the polymers are still present as shown by the saturated detachment length. To understand this decrease in adhesive work on the surface measurement above 10 wt%, we measure the interaction of the tip, specifically the indentation, as a function of additive concentration. Adhesive work elucidates the adhesive properties of the layer and indentation reveals more information on the local stiffness of the layer. From the force–distance curves at 1 wt% (Fig. 2e and f) we can already see that the indentation increases when we move closer to the edge, as the layer becomes softer by the presence of the additive as indicated by the vertical lines. Both additives show an increase in indentation as we move away from the bulk towards the edge.

**Fig. 4:**
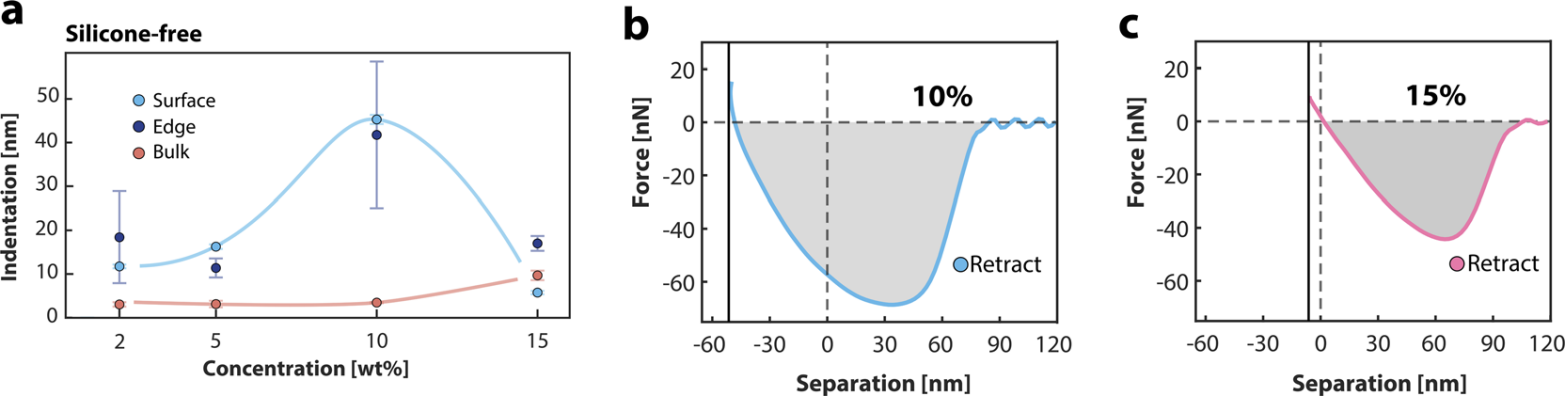
(a) Indentation of the different polymer films in the bulk (orange), edge (blue) and surface (light blue). (b) Force–distance retract curve measured at the surface of the 10 wt% film. (c) Surface force–distance retract curve measured at the surface of the 15 wt% film, displaying a lower indentation compared to the 10 wt% film.

Fig. 4a shows the indentation of the films as a function of additive concentration. On the surface, the indentation increases as we increase the amount of additive up to 10 wt% where it reaches a maximum. The edge and surface indentation are very comparable, indicating similar surface functionalisation measured in cross-sectional and surface spectroscopy. Between 10 and 15 wt%, the indentation on the surface and edge drops accompanied by an increase in bulk indentation. Roughness measurements indicate a RMS value of ≈9 nm at the edge and ≈6 nm for the bulk of the 10 wt%-film and ≈18 nm at the edge and bulk of the 15 wt%-film.

For clarity, we provide the force–distance curves, recorded on the surfaces of the films containing 10 wt% (Fig. 4b) and 15 wt% (Fig. 4c), showing the decrease of the indentation at 15 wt%. When we consider the relative differences between the measured properties, there are multiple explanations possible.

On a macroscopic scale, the simultaneous decrease in adhesive work and indentation observed above 10 wt% (cross-sectional, Fig. 4a, and surface, inset Fig. 3e) indicates that the layer becomes stiffer and less adhesive. Because the adhesive work is highly sensitive to long-range tip–sample interactions, and capillary and polymeric contributions cannot be separated in our data, our data can therefore be interpreted in several, but not mutually exclusive, ways: (i) a change in surface energy that changes the capillary meniscus and therefore the measured adhesive work. (ii) A structural transition from an isotropic-like to a more nematic-like ordering of the surface chains, leading to a tighter, more interwoven polymer matrix and thus reduced indentation.^38,39^ (iii) An increase in crosslink density between the additive and the base polymer matrix,^40^ which further stiffens the film and suppresses polymer–tip bridging. Finally, we would like to emphasise that increasing the additive concentration beyond 10 wt% results in more assimilated polymers in the bulk, and the whole film becomes saturated with the additive. Bulk adhesive properties will increase, but edge and surface properties remain constant due to the surface saturation of the film. Higher concentrations are beyond the range of industrial formulations for flexographic printing processes and were therefore not studied further.

## 4 Conclusions

We investigated the impact of additives on polymeric thin films used in printing processes like flexographic printing. In flexographic metal printing, 2D metallic flakes are transferred to a polymer film to create a patterned metallic surface. After measuring the polymer film without additives, we used 2 types of additives to investigate the modified properties of the film due to the additive presence. Using AFM, we analyzed the mechanical properties and adhesive characteristics of films with varying additive concentrations. Cross-sectional AFM revealed that the silicone additive reduces the adhesive properties of the film, while the silicone-free additive improves the adhesive properties. Both types of additives segregate towards the film surface, with adhesive work and detachment length increasing up to 10 wt%, beyond which saturation occurs. Surface measurements indicate a drastic change in the film's properties between 10 wt% and 15 wt%.

## Author contributions

IW created, and measured the surface tension of the polymer samples together with JA in Lehrte. SH prepared the samples for cross-section measurements and performed the experiments. SH wrote the manuscript, and IW, PB, JA and HJWZ revised the manuscript. PB supervised the project.

## Conflicts of interest

The authors state that there are no conflicts to declare.

## Supplementary Material

NA-OLF-D5NA00956A-s001

## Data Availability

Data supporting the results of this article are published and openly available at the following URL/DOI: https://doi.org/10.4121/9D5817D4-B63F-42E9-A97B-678285D6F56D.^41^ Supplementary information (SI) is available. See DOI: https://doi.org/10.1039/d5na00956a.
