# Supplementary material for: Tailoring the adhesion properties of thin polymeric films using additives: an AFM study
Source: Nanoscale Adv. 2026 Jan 14;8(4):1273–80. doi: 10.1039/d5na00956a (PMC12801337; doi:10.1039/d5na00956a)
Supplement: NA-008-D5NA00956A-s001 [file NA-008-D5NA00956A-s001.pdf]

Supplementary Information (SI) for Nanoscale Advances.
This journal is © The Royal Society of Chemistry 2026

Supporting Information to the article Tailoring the adhesion properties of thin polymeric films using additives: an AFM study.

Sebastiaan Haartsen,[1] Johannes Aprojanz,[2] Inga Wille,[2] Harald Jasper,[2] Harold Zandvliet,[1] and P. Bampoulis[1]

[1)]*Physics of Interfaces and Nanomaterials group, MESA+ Institute for Nanotechnology and University of Twente, PO Box 217, 7500AE Enschede*

[2)]*ACTEGA Metal Print GmbH, Mielestrasse 13, 31275 Lehrte, Germany.*

(Dated: 11 December 2025)

Supplementary information supporting the article: Tailoring the adhesion properties of thin polymeric films using additives: an AFM study. In this document, we detail our sample fabrication method for cross-sectional analysis, we discuss our calibration procedure and measurements. Next we display the stiffness, indentation and adhesion of a polymer film with saturated stiffness measurements that were omitted from the article. Finally we detail our procedure for detecting the edge of the polymer film.

I. SAMPLE FABRICATION

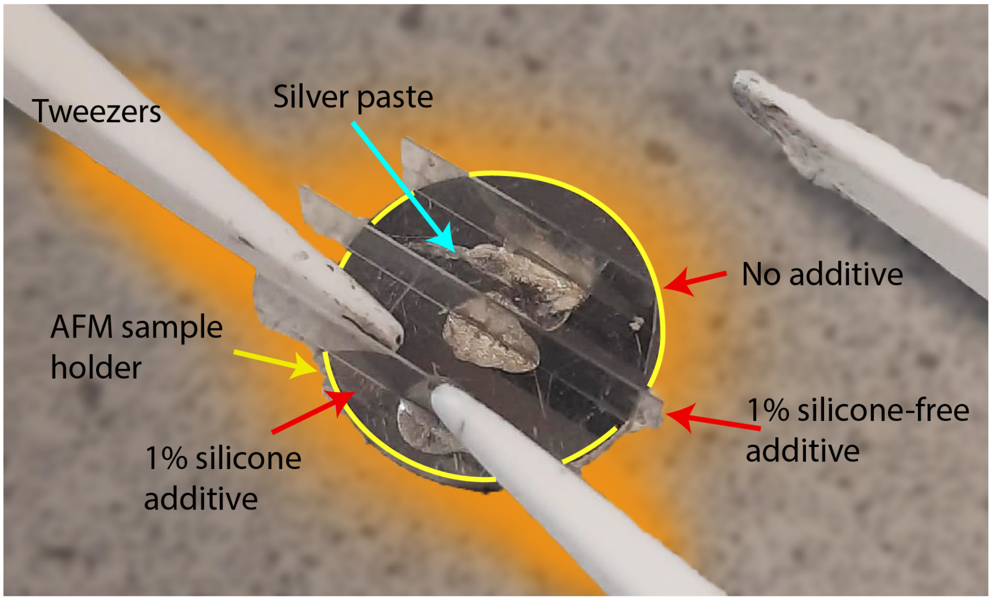

FIG. 1. Photo of the sample showing 3 samples used for crossectional AFM-measurements.

The polymer mixture is prepared in liquid form according to the specifications in the main paper. We use 2 monomers a long- and a short aliphatic polyether diacrylate along with a type 1 and type 2 photoinitiator mix to create the base polymer mixture. The liquid additive is added after preparation of the base mixture using a balance to ensure the correct concentration. The entire mixture is stirred and heated to 50 °C for ≈10 minutes. Using a pipette, a droplet of the mixture is placed on a polyethylene terephthalate (PET) substrate and spread using a 12 µm-rollerbar. After UV curing the polymer film hardens and sticks to the PET surface. The samples are cut using IPA-cleaned scissors and placed on the magnetic AFM sample holder shown in figure 1. Using silverpaste and tweezers for structural support (see figure 1), the samples are glued on the holder. After about 10 minutes, the glue has hardened, and the sample holder is placed in the AFM setup for cross-sectional measurements. The stiffness of the PET substrate provides a solid structure to ensure that the polymer film is aligned in the correct position. The sample is rotated so the AFM tip can approach the sample perpendicular to the roller direction, which can be fine tuned in the AFM control software.

II. AFM TIP CALIBRATION

All measurements were performed using the PPP-FMR-probes from Nanosensors, with a nominal spring constant: $C = 4\,N\,m^{-1}$, resonance frequency: $f = 75\,kHz$ and a radius of curvature at the apex of 7 nm. The individual probes' mechanical properties are calibrated using a stiff sapphire sample. We measure the spring constant before and after measurements on the polymer films. The average spring constant of the AFM-probe is $3.845\,N\,m^{-1}$ with a deviation of $\pm 0.137\,N\,m^{-1}$ for all used probes. Due to a high probability of polymers attaching to the AFM tip, an additional calibration step is added after measurements on the sapphire. The polymeric interactions due to polymers attached to the tip can be identified using force-distance analysis due to additional tip-interactions[1]. We perform multiple high-resolution force-distance curves on a calibration sample with known mechanical properties to identify probe interactions. Figure 2 shows force-distance

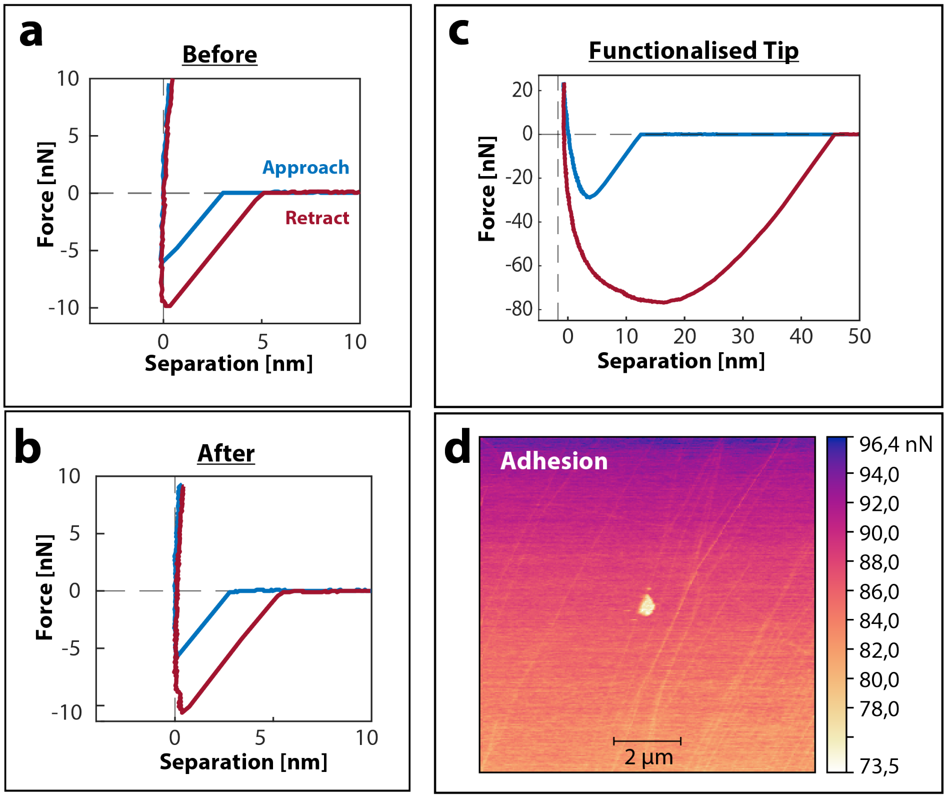

FIG. 2. **(a)** Force distance curve measured on HOPG before measurements on a polymer film. **(b)** Force distance curve measured on HOPG of a tip with recoverable properties. **(c)** Force distance measurement on HOPG of a tip with polymers attached. **(d)** Adhesion map of HOPG, measured with a functionalised tip.

curves and an adhesion map measured on cleaved HOPG in ambient conditions. 2a shows a force-

distance curve recorded with a driving frequency of 1 Hz before measuring a polymer films, 2b and c show force-distance curves measured after use on a polymer sample. Differences between 2a and b are minimal, and the probe's mechanical properties are retained. The linear force dependence in the retract curve is explained by the formation of a liquid bridge between the tip and sample. As the tip retracts, the bridge breaks and the capillary force reduces[2].

Figure 2c shows a clear change in the probe's mechanical properties. The larger Van der Waals force in the approach curve and adhesive force in the retract curve indicate a larger probe due to tip blunting or attached polymers. Additionally, the shape of the curve indicates long-range interactions that detail an attractive force between the tip and sample from polymers that attach to the surface and detach during retraction. The large number of interacting polymers results in a long-range non-linear interaction, typical for tip-polymer interactions. Attached polymers, due to continued scanning on the polymer films, significantly increase the contact area of the AFM-tip. The change in the probe interactions is reflected in a direct increase in the measured adhesive force by several multiples, and a noticeable contrast change in the scanned area. figure 2d displays an adhesion map, measured on HOPG. The area is scanned top to bottom, left to right. The adhesion gradient visible from the top to the bottom indicates a decrease in adhesive properties when as a result of polymers detaching from the tip.

We continue scanning on HOPG until force-distance analysis measurements show no evidence of polymer interactions, and we recover a similar curve as seen in figure 2b. The cleaved HOPG creates a clean surface without polymers. Polymeric interactions we measure on the cleaved HOPG must be from polymers attached to the tip. The measured adhesion and shape of the force-distance curve from the HOPG sample are used to calibrate and determine whether the probe's interactions depend solely on contact and capillary forces.AFM-probes without polymer interactions are used to measure the mechanical properties of the film to ensure that polymer interactions are depend solely on polymers from the polymer film and not the AFM-probe. Polymer attachment during scanning is possible and is detected by a contrast change during the measurement. Following such an event, the film measurement is stopped, and the tip is cleaned on cleaved HOPG.

III. STIFFNESS MEASUREMENTS

The stiffness measurements were omitted from the article due to inaccurate responses from the measurement setup. The difference with respect to the indentation and adhesion measurement is

shown in figure 3. During measaurements the calculated stiffness saturated and caused images

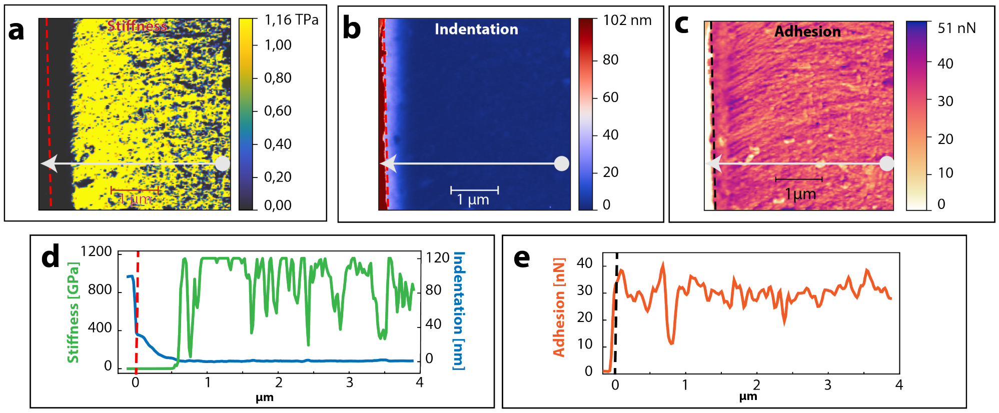

FIG. 3. **(a)** Stiffness, **(b)** indentation and **(c)** adhesion maps with their respective **(d,e)** line profile. All maps are measured simultaneously. The edge (dotted line) is clearly visible in the indentation map, but is lost due to saturated values in the stiffness map, resulting in an inaccurate description of the edge.

with an inaccurate contrast and unusable data. The indentation of the material is connected to the stiffness of the material since both properties are determined from force-distance analysis when the tip is pushed into the sample. As opposed to the 'tail'-section, where the tip is pulled from the sample.

IV. EDGE DETECTION

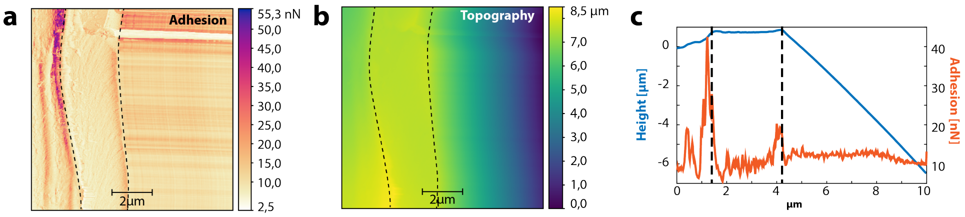

FIG. 4. **(a)** Topography and **(b)** adhesion maps of a 3 µm-thin polymer film. **(c)** Line scan of the topography and adhesion to determine the edges of the polymer sample.

Cross-sectional measurements on these samples cause the apex of the tip to lose contact when the measurement takes place beyond the film's surface. After the apex of the tip loses contact, the side of the tip, is still in contact and we map the tip geometry instead of the sample topography. This is reflected by elongated stripes in the tip-movement direction (left to right) and a drop in

measured sample height. Figure 4a, b shows the topography and adhesion map of a 3 µm-film. The lines indicate the interfaces of the polymer film. Left of the line, we measure on the PET-substrate, right of the right line, the apex of the tip is out of contact. The low adhesive (white) feature in the top is elongated from left to right as the side of the tip measures the same local adhesive interactions for the entire length. The edge is determined from both measurements. The line scan in figure 4 highlights the constant adhesion in comparison to the drop in height.

REFERENCES

[1]H. Zeng, J. Huang, Y. Tian, L. Li, M. V. Tirrell, and J. N. Israelachvili, "Adhesion and Detachment Mechanisms between Polymer and Solid Substrate Surfaces: Using Polystyrene-Mica as a Model System," Macromolecules **49**, 5223–5231 (2016).

[2]Z. Wei and Y. P. Zhao, "Growth of liquid bridge in AFM," Journal of Physics D: Applied Physics **40**, 4368–4375 (2007).
